# Role of Ivabradine in Patients with Heart Failure with Preserved Ejection Fraction

**DOI:** 10.7759/cureus.7123

**Published:** 2020-02-27

**Authors:** Muhammad Nadeem, Mohab Hassib, Hafiz M Aslam, Dania Fatima, Yasir Illahi

**Affiliations:** 1 Internal Medicine, Seton Hall University, St. Francis Medical Center, Trenton, USA; 2 Internal Medicine, Saint Francis Medical Center, Trenton, USA; 3 Internal Medicine, Seton Hall University, Hackensack Meridian School of Medicine, Trenton, USA; 4 Internal Medicine, Nishtar Medical College, Multan, PAK; 5 Internal Medicine, University of Toledo, Toledo, USA

**Keywords:** heart failure, ivabradine

## Abstract

Heart failure with preserved ejection fraction (HFpEF) accounts for 50% of patients with heart failure. HFpEF carries almost similar morbidity and mortality outcomes to heart failure with reduced ejection fraction (HFrEF). Despite many trials, no management has been shown to improve mortality outcomes in HFpEF. An elevated heart rate in patients with HFpEF has been associated with worse outcomes. Previous trials on the use of beta-blockers in reducing the heart rate in patients with HFpEF have shown worse outcomes, possibly due to the negative inotropic effects. The funny current inhibitor, ivabradine, results in a reduced heart rate without affecting inotropy. Two randomized controlled trials and one cross-over study have evaluated the use of ivabradine in HFpEF patients. The outcomes of the trials have been heterogeneous; ivabradine showed improved exercise tolerance, no change in primary endpoints was seen l, and there was a worsening in the outcomes. Our review underscores the requirement of a large randomized clinical trial in the appropriate patient population.

## Introduction and background

Heart failure with preserved ejection fraction (HFpEF) accounts for 50% of patients with heart failure, and its prevalence is rising with an aging population [1]. Criteria for the diagnosis of HFpEF based on the European Society of Cardiology’s guidelines include the presence of symptoms and signs, left ventricular ejection fraction more than or equal to 50%, an elevated N-terminal pro-b-type natriuretic peptide (NT-pro-BNP) or BNP, and either of the following: relevant structural left heart disease like left ventricular hypertrophy (LVH) or left atrial enlargement (LAE) or evidence of diastolic dysfunction. Mortality outcomes of patients with HFpEF are similar to mortality associated with patients with HFrEF [2]. There have not been any management strategies that reduce morbidity or mortality in HFpEF.

The pathophysiology of heart failure with preserved ejection fraction is multifactorial and includes systemic inflammation, left ventricular (LV) hypertrophy, slow LV relaxation, LV diastolic stiffness, decreased LV systolic performance, left atrial remodeling, peripheral vascular resistance, impaired epithelial function, increased pulmonary arterial and venous resistance, neurohormonal activation, and ventricular-arterial coupling. The maladaptive response between arterial elastance and ventricular systolic elastance, called ventricular-arterial coupling, is one of the theories proposed by Borlaug [3]. In those with pre-existing vascular dysfunction, when combined with afterload elevation and ventricular-arterial stiffening, it can lead to an increase in blood pressure [4]. This mechanism then impairs diastolic LV relaxation, leading to further increases in blood pressure that can contribute to dramatic increases in LV filling pressures during stress. This adaptive physiologic response evolves into a pathologic response such as incident HFpEF. Hypertension (HTN) or elevated blood pressure is a well-documented antecedent to the development of HF; as many as 90% of heart failure (HF) cases are preceded by the diagnosis of HTN. In the Efficacy in Diastolic Dysfunction (EXCEED) trial, 527 patients with early-stage HTN, after 24-48 weeks of antihypertensive therapy, showed evidence of an increase in the effective Ea/Ees ratio (r = -0.25, p= 0.01) [5]. This change in Ea/Ees ratio due to anti-hypertensive therapy in patients with diastolic dysfunction and hypertension suggests that anti-hypertensive therapy could help prevent the development of hypertensive heart disease and, later, decompensation to HFpEF. There are two mechanisms responsible for diastolic dysfunction: abnormal relaxation of the LV and decreased compliance of the LV. Echocardiography in these patients shows prolonged isovolumetric relaxation, slowed LV filling, and increased diastolic stiffness. The primary markers of diastolic dysfunction are mitral inflow, which includes peak early filling (E-wave) and late diastolic filling (A-wave) velocities, and the E/A ratio. According to the European Society of Cardiology Guidelines, early mitral valve annular velocity (e’) and the ratio of E (early ventricular filling)/e’(early mitral valve annular velocity) are important in estimating LV filling pressures [6]. An E/e’ of 15 is diagnostic of LV diastolic dysfunction (DD) and an E/e’ <8 excludes HFpEF. E/e’ values ranging from 8 to 15 are suggestive of LV DD that requires further evaluation with biomarkers such as E/A ratio or BNP. The renin-angiotensin-aldosterone system (RAAS) also has a role in the pathophysiology of HFpEF. This is due to the role of hypertension in the pathogenesis of HFpEF and RAAS, in turn, being responsible for systolic hypertension and the remodeling of the myocardium [7-9].

An elevated heart rate has been associated with worse prognostic outcomes in those with heart failure with preserved ejection fraction [1]. Early on in their disease, patients with HFpEF have impaired LV relaxation rather than impaired LV compliance or raised filling pressures in the resting state. Higher heart rates during exercise may be detrimental owing to reducing the time for diastolic filling and, in turn, causing increased LV filling pressures and reduction in exercise tolerance. Prolonging the diastolic phase may result in an improvement in exercise tolerance [10]. One randomized controlled trial involving beta-blockers in patients with HFpEF did not show an improvement in cardiovascular mortality or HF hospitalizations [11]. Beta-blockers have rather been associated with worsening diastolic dysfunction [12]. The resultant negative inotropy may be responsible for the failure to improve exercise tolerance and peak oxygen uptake [13]. Ivabradine decreases the heart rate by selectively inhibiting the sino-atrial node’s funny current channels [14]. Ivabradine has been associated with a reduction in all-cause mortality, cardiovascular mortality, and heart failure hospitalizations in patients with HFrEF [9]. Ivabradine might also have a role in patients with HFpEF, as there is no negative inotropy associated with this drug. There have only been two randomized trials and one cross-over study on the role of ivabradine in the setting of HFpEF. Our review will focus on the effects of ivabradine on exercise tolerance, peak oxygen uptake, echocardiographic parameters, hemodynamics, and BNP levels.

## Review

Effect on impaired exercise tolerance

Evidence of decreased exercise tolerance is usually the first sign of early HFpEF. Systolic and diastolic reserves are impaired in patients with HFpEF, and exercise may reveal these mild defects. Impaired diastolic filling during exercise results in elevated filling pressures. This manifests in an elevated E/e' ratio, which contributes to exertional dyspnea. The role of ivabradine in decreasing the heart rate and possibly improving exercise tolerance has been studied in three randomized clinical trials (RCTs). Kosmala et al. conducted a randomized controlled trial that included 61 patients who received either Ivabradine or a placebo for seven days before a follow-up assessment [10]. Baseline measures of exercise tolerance (METS on treadmill and peak oxygen uptake) were taken. The group that was given ivabradine showed a significant improvement in exercise capacity (METS baseline Iva group vs METS follow-up Iva group p=0.001 and peak VO2 baseline Iva group vs peak VO2 follow-up Iva group p=0.001) versus the control group (METS baseline control group vs METS follow-up control group p=0.35 and peak VO2 baseline control group vs peak VO2 follow-up control group p=0.54). Changes in METS in the treated group were greater than in the control group (1.5+/-1.2 vs 0.4+/-1.2 p=0.001) as were changes in peak VO2 (3+/-3.6 ml/kg/min vs 0.4+/-2.7 ml/kg/min p=0.003). However, Komajda et al. published a randomized controlled trial in 2017 that included 179 patients with HFpEF (and was based on long-term follow-up (eight months)) that showed no significant change in exercise tolerance [1]. The six-minute walk (6MWT) test did not differ on follow-up examination in the group that received ivabradine (baseline median 6 MWT 323 m (Q1-Q3 243.5-375.0) and change in median 6 MWT (last post-baseline value from baseline) 0.0 m (Q1-Q3 -28.5-35.0)). A comparison of the post-baseline median 6 MWT values did not show significant differences between the two groups (p=0.882). In addition, most patients (78.2% vs 83.3%) did not show any improvement in their New York Heart Association (NYHA) class in the last post-baseline assessment. There is one placebo-controlled, cross-over clinical study conducted by Pal et al. that evaluated the short-term role of ivabradine in HFpEF patients [2]. Paired comparisons of the changes in peak VO2 resulting from the two-week intervention blocks demonstrated a decrease in VO2 in the ivabradine group (−2.1 versus 0.9 mL·kg−1·min−1; P=0.003) and significantly reduced submaximal exercise capacity. A fixed dose of 5 mg of ivabradine was used in the trial conducted by Kosmala et al., rather than titration of the dose to reach a specific heart rate. Follow-up assessment might have resulted in less chronotropic incompetence as compared to the titrated heart rate in other RCTs. Patients enrolled in the RCT by Komajda et al. may have had advanced HFpEF with extensive myocardial fibrosis that could have caused the failure of the pharmacological intervention. Extensive myocardial fibrosis results in a severe reduction in stroke volume. This, in turn, makes cardiac output dependent on the heart rate, and reduction of the heart rate in this setting would be detrimental. It is difficult to differentiate between HFpEF with a restriction defect and HFpEF with a relaxation defect. Pal et al.’s cross-over study included patients with chronotropic incompetence, poor stroke volume reserve, and heart rate reduction, which resulted in worsening exercise tolerance.

Effect on echocardiographic parameters

There was a significant improvement in the resting LV lusitropic function indicated by higher septal e’ in the treated group (5.4 +/- 1.1 vs 6.1 +/- 1.3 p=0.007). There was no evidence that a change in septal velocity occurred in response to increased preload; this was recognized because no change was seen in the resting E/e’ ratio. However, in the post-hoc analysis, this effect was only seen in patients with grade I diastolic dysfunction (E/A ratio 0.75 +/- 0.12 at baseline vs. 0.94 +/-0.26 at follow-up, p=0.01, and septal e’ 5.2 +/- 1.1 cm/s at baseline vs. 6.0 +/- 1.4 cm/s at follow-up, p=0.004) but not in patients with diastolic dysfunction of grade II or higher (E/A ratio 1.42 +/- 0.50 at baseline vs. 1.50 +/- 0.43 at follow-up, p=0.83, and septal e’ 6.0 +/- 1.2 cm/s at baseline vs. 6.2 +/- 1.0 cm/s at follow-up, p= .61) (Kosmala et al. [10]). The treatment group showed a significant reduction in the exercise-induced E/e’ ratio. The clinical trial conducted by Komajda et al. showed no significant change in E/e’ in both the treated group, as well as when compared between treatment and placebo groups (p=0.135 between groups analysis) [1]. Pal et al. found a mild but significant increase in the E/A ratio in the ivabradine-treated HFpEF cohort (0.60 vs 0.65 p=0.026) [2]. There was also an increase in the mean e’ velocity (4.5 +/- 1.1 vs 5.4 +/- 1.5 p=0.002), however, there was no effect seen on the E/e’ ratio (10.4 +/- 2.5 vs 10.7 +/- 2.4 p= 0.56). It could be construed that the patient population included in Kosmala et al.’s RCT may have had HFpEF without extensive myocardial fibrosis, indicated by the significant change in the septal e’. The possibility of extensive myocardial fibrosis in the patient population may have resulted in a severe reduction in stroke volume, making cardiac output dependent on heart rate. Reduction in the heart rate in this setting would again be detrimental. Pal et al.’s cross-over study included patients with poor stroke volume reserve and heart rate reduction. This resulted in worsening exercise tolerance, despite mild changes in the E/A ratio as well as mean e’ velocity.

Effect on NT-pro-BNP or BNP

The RCT by Kosmala et al. showed no significant changes in the BNP levels in the subgroup analysis (grade I diastolic dysfunction Iva group baseline and follow-up p=0.63, grade ≥ II diastolic dysfunction Iva group baseline and follow-up p=0.30, grade I Iva gp vs grade II diastolic dysfunction Iva gp p=0.23) [10]. There were no changes in baseline versus follow-up values of BNP in the placebo cohort when sub-group analysis was done for all grades of diastolic dysfunction. Similarly, there were no significant differences seen in the sub-group analysis in those with a heart rate of less than 70 versus those with a heart rate of more than 70 as well as patients with a normal chronotropic heart rate response versus patients with chronotropic incompetence. Komajda et al. showed no difference in the median follow-up value of NT-pro-BNP between ivabradine and placebo groups (1.01, 90% confidence interval -0.86 to 1.19; P =0.882) [1]. Kosmala et al. tested only the short-term role of ivabradine in patients with HFpEF. This might have precluded the evaluation of remodeling and may have been the reason that there were no changes found in BNP levels. However, there were no significant changes in NT-pro-BNP in Komajda et al.’s trial. Inadequate sample size may have also contributed to these findings.

Effect on hemodynamics

There was a decrease in the resting heart rate in the treatment group. The absence of changes in the heart rate in response to exercise may be reflective of differences in exercise performance. Workload corrected chronotropic response (WCCR), defined as the difference in heart rate at the same exercise time on the baseline and follow-up tests, showed a slower increase in heart rate during exercise in the treated group (Kosmala et al.) [10]. Ivabradine reduced the mean resting heart rate by 20 bpm (77 to 57 bpm; P<0.0001) without affecting blood pressure or left ventricular ejection fraction (EF). Ivabradine was also shown to reduce the chronotropic response to exercise (peak heart rate 129 versus 107 bpm; P<0.0001) (Pal et al. [2]). The heart rate reduction was accompanied by reduced peak oxygen consumption in the majority of HFpEF patients (19 patients had a reduction in the Vo2 peak) with a diminution in the VO2 peak from 15.9 to 14.8 mL·kg^−1^·min^−1^ (P=0.003) without significantly affecting VE/ VCO2 slope or the anaerobic threshold. Moreover, a paired comparison of the changes in VO2 peak resulting from the two-week intervention blocks demonstrated a decrease in VO2 in the ivabradine group (−2.1 versus 0.9 mL·kg^−1^·min^−1^; P=0.003). Most of the patients included had chronotropic incompetence as well as poor cardiac output reserve, which might have contributed towards worsening peak oxygen uptake and exercise tolerance.

## Conclusions

There are several factors that contributed toward the significant change in peak oxygen uptake, exercise tolerance, and septal e’ velocity in patients in the RCT conducted. First, a fixed dose of 5 mg of ivabradine was used without titration to a specific heart rate, which may have resulted in less chronotropic incompetence. Second, patients without myocardial fibrosis might have been represented in the cohort, which leads to an improvement in exercise tolerance indicated by a decrease in the E/e’ ratio (left ventricular filling pressure) post-exercise. RCT failed to show an improvement in exercise tolerance and peak oxygen uptake. There is a possibility that most of the patients in this study had extensive myocardial fibrosis, resulting in decreased cardiac output and a reduction in the heart rate. This may have also contributed to a worsening of the exercise tolerance in these patients. The RCT had a population similar to patients and showed a worsening of exercise tolerance with ivabradine. Moreover, limited sample sizes in all three trials precluded the application of multivariate regression analysis. Our review underscores the need for large-scale RCTs for the role of ivabradine in HFpEF patients for both short-term and long-term outcomes, especially with regards to morbidity and mortality. The trial must include baseline exercise tolerance testing to quantify chronotropic incompetence, baseline cardiac MRI, for evaluation of myocardial fibrosis, and different dosage arms to evaluate ivabradine in patients with HFpEF.
